# Supplementary material for: Identification of the Novel Oncogenic Role of SAAL1 and Its Therapeutic Potential in Hepatocellular Carcinoma
Source: Cancers (Basel). 2020 Jul 8;12(7):1843. doi: 10.3390/cancers12071843 (PMC7408781; doi:10.3390/cancers12071843)
Supplement: Supplementary file 1 [file cancers-12-01843-s001.zip › cancers-864954-suppl/cancers-864754-supplemantary figures and tables.docx]

Article

Identification of the Novel Oncogenic Role of SAAL1 and Its Therapeutic Potential in Hepatocellular Carcinoma

Pei-Yi Chu, Shiao-Lin Tung, Kuo-Wang Tsai, Fang-Ping Shen and Shih-Hsuan Chan

Supplementary Materials


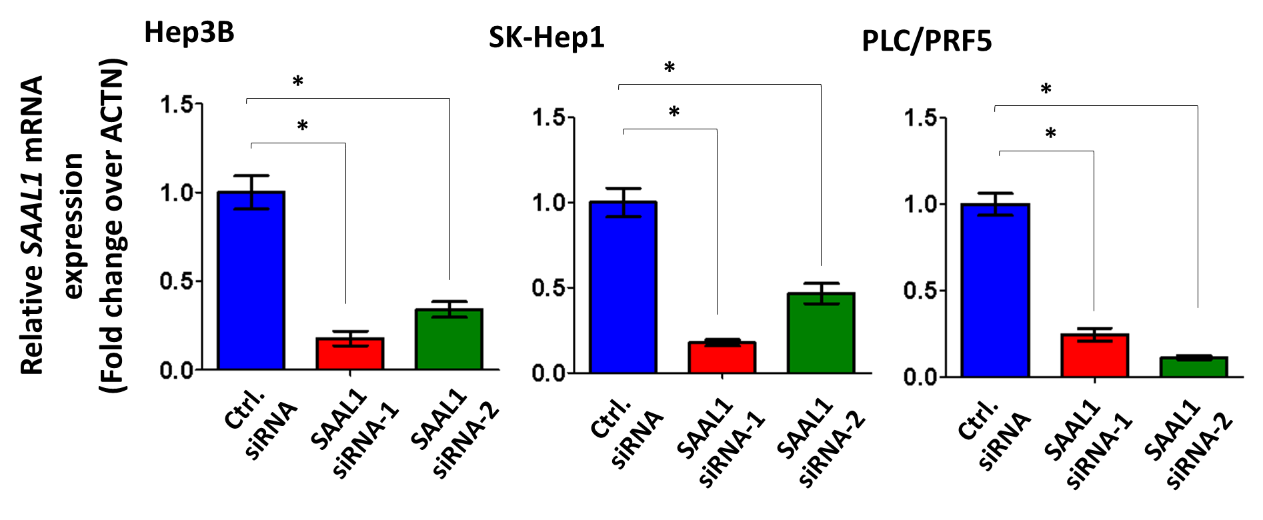


**Supplementary Figure S1.** qRT-PCR analysis of SAAL1 siRNA Knockdown efficiency in three HCC cancer cell lines. SAAL1 mRNA expressions were normalized to Actin mRNA expressions. Data are means ± SEM (*n* = 3). * *p* < 0.05.


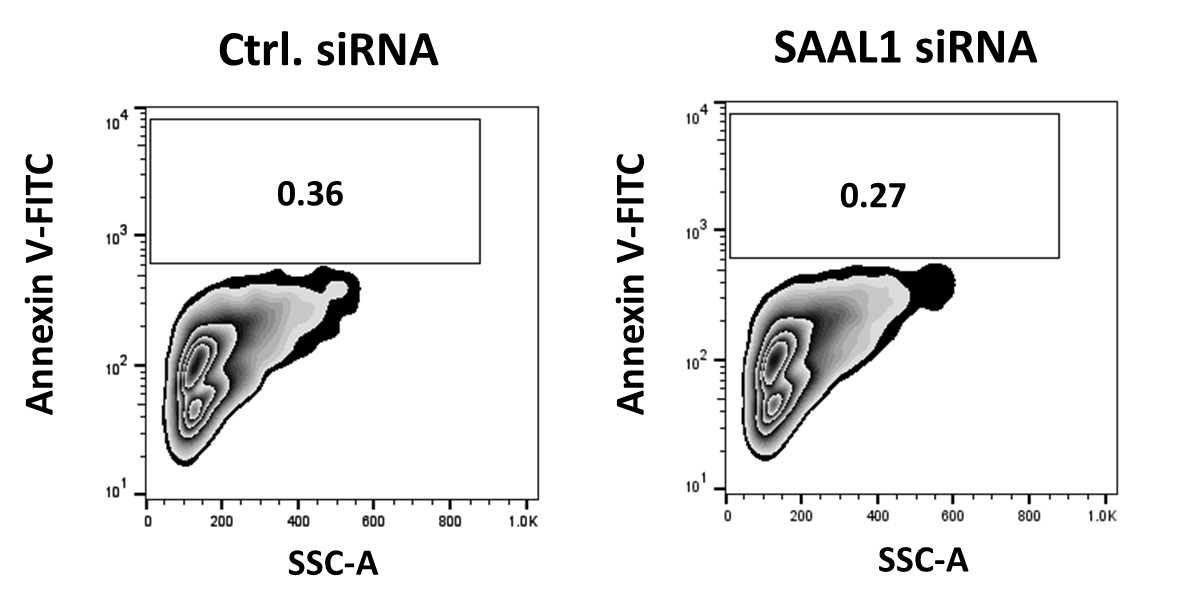


**Supplementary Figure S2.** Flow cytometry analysis of annexin V expression in SAAL1-depleted SK-Hep1 cells. The SK-Hep1 cells were transfected with the control and SAAL1 siRNA respectively for 48 hours followed by annexin V-FITC staining. The representative figures were shown.


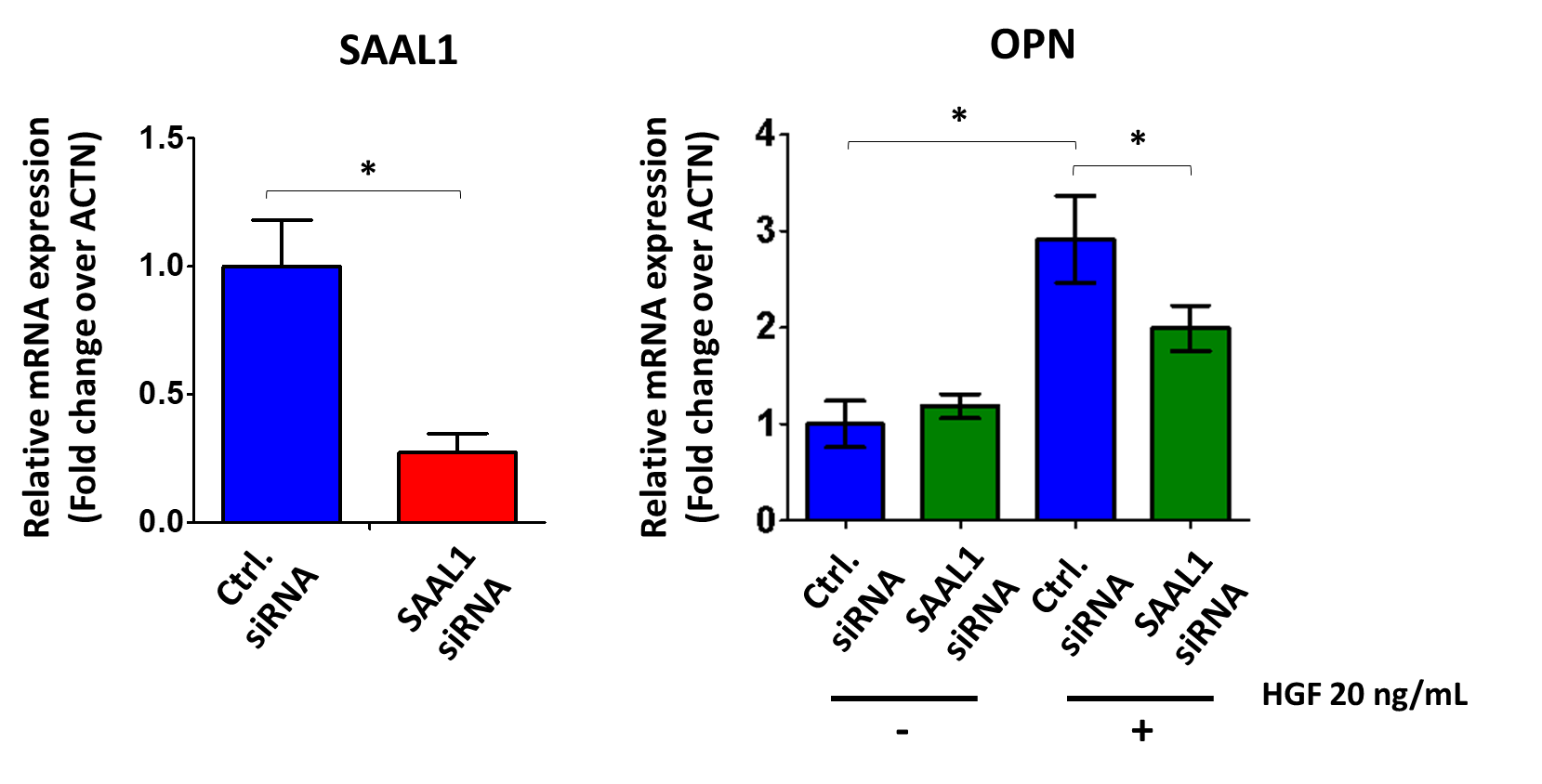


**Supplementary Figure S3.** SAAL1 depletion significantly abolishes HGF-induced Osteopontin (OPN) expression. Knockdown efficiency of SAAL1 siRNA and HGF-induced OPN expression were measured by qRT-PCR. Data are means ± SEM (*n* = 3). * *p* < 0.05.

**Supplementary Table S1.** Correlation between expression levels of SAAL1 and clinicopathologic features of HCC patients.

| **Variables** | SAAL1 high (*n* = 151) | SAAL1 low (*n* = 195) | *p-*value ^a^ |
| --- | --- | --- | --- |
|  | Number (%) | Number (%) |  |
| **AJCC Pathological stage** |  |  |  |
| I, II | 108 (71.5) | 148 (75.9) | 0.388 |
| III, IV | 43 (28.5) | 43 (24.1) |  |
| **pT stage** |  |  |  |
| T1, T2 | 111 (73.5) | 149 (76.4) | 0.616 |
| T3, T4 | 40 (26.5) | 46 (23.6) |  |
| **pN stage** |  |  |  |
| N0 | 148 (98.0) | 194 (99.5) | 0.321 |
| N1 | 3 (2.0) | 1 (0.5) |  |
| **pM stage** |  |  |  |
| M0 | 148 (98.0) | 194 (99.5) | 0.321 |
| M1 | 3 (2.0) | 1 (0.5) |  |

^a^ *p-*value is estimated by Fisher’s exact test.

**Supplementary Table S2.** The detailed information of antibodies, reagents, chemicals and small interference RNA (siRNA) used in this study.

| **Antibodies and Reagents** | **Vendor** | **Catalog Number** |
| --- | --- | --- |
| Anti-Met antibody | Cell Signaling Technology, Danvers, MA, USA | 8198S |
| Anti-SAAL1 antibody | Bethyl Laboratory Inc., Montgomery, TX, USA | A304-966A |
| Anti-p-Met antibody | Cell Signaling Technology, Danvers, MA, USA | 3077S |
| Anti-Akt antibody | Cell Signaling Technology, Danvers, MA, USA | 4691S |
| Anti-p-Akt antibody | Cell Signaling Technology, Danvers, MA, USA | 9271S |
| Anti-mTOR antibody | Cell Signaling Technology, Danvers, MA, USA | #2983 |
| Anti-p-mTOR antibody | Cell Signaling Technology, Danvers, MA, USA | #2974 |
| Anti-β-actin antibody | Santa Cruz Biotechnology, Santa Cruz, CA, USA | sc-47778 |
| Anti-α-Tubulin antibody | Santa Cruz Biotechnology, Santa Cruz, CA, USA | sc-5286 |
| Anti-NA.K ATPase antibody | Cell Signaling Technology, Danvers, MA, USA | 23565 |
| Anti-Lamin B2 antibody | Abcam, San Francisco, CA, USA | Ab151735 |
| HGF recombinant protein | R＆D systems, MN, USA | 294-HG |
| *Trans*IT-X2® System | Mirus, Madison, WI, USA | MIR6000 |
| FITC annexin V | BD Biosciences | 51-65874X |
| propidium iodide | Sigma-Aldrich | P4170 |
| **siRNA** | **Oligonucleotide sequences used for siRNA**  **Transfection** | **Vendor** |
| Control siRNA | CCTAAGGTTAAGTCGCCCTCG | MDBIO, Taiwan |
| SAAL1 siRNA-1(448-467) | CCACCUACUCUGCUGGAAATT | MDBIO, Taiwan |
| SAAL1 siRNA-2(615-634) | GGUUGUGGACAAGCUCUUUTT | MDBIO, Taiwan |

| 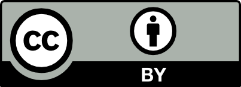 | © 2020 by the authors. Licensee MDPI, Basel, Switzerland. This article is an open access article distributed under the terms and conditions of the Creative Commons Attribution (CC BY) license (http://creativecommons.org/licenses/by/4.0/). |
| --- | --- |
