# Supplementary figures and images for: Identification of the Novel Oncogenic Role of SAAL1 and Its Therapeutic Potential in Hepatocellular Carcinoma
Source: Cancers (Basel). 2020 Jul 8;12(7):1843. doi: 10.3390/cancers12071843 (PMC7408781; doi:10.3390/cancers12071843)

Figure 2A

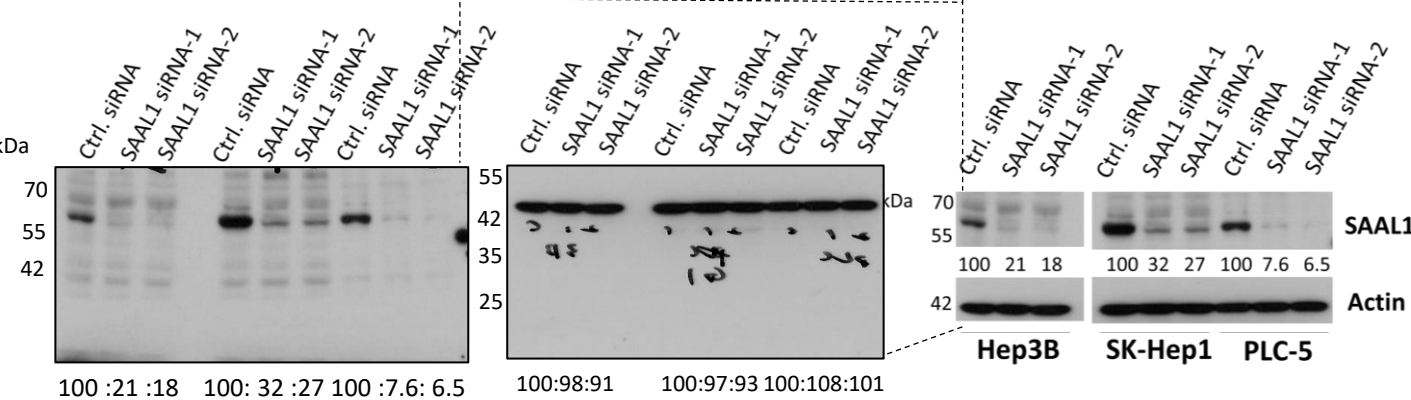

Figure 4A

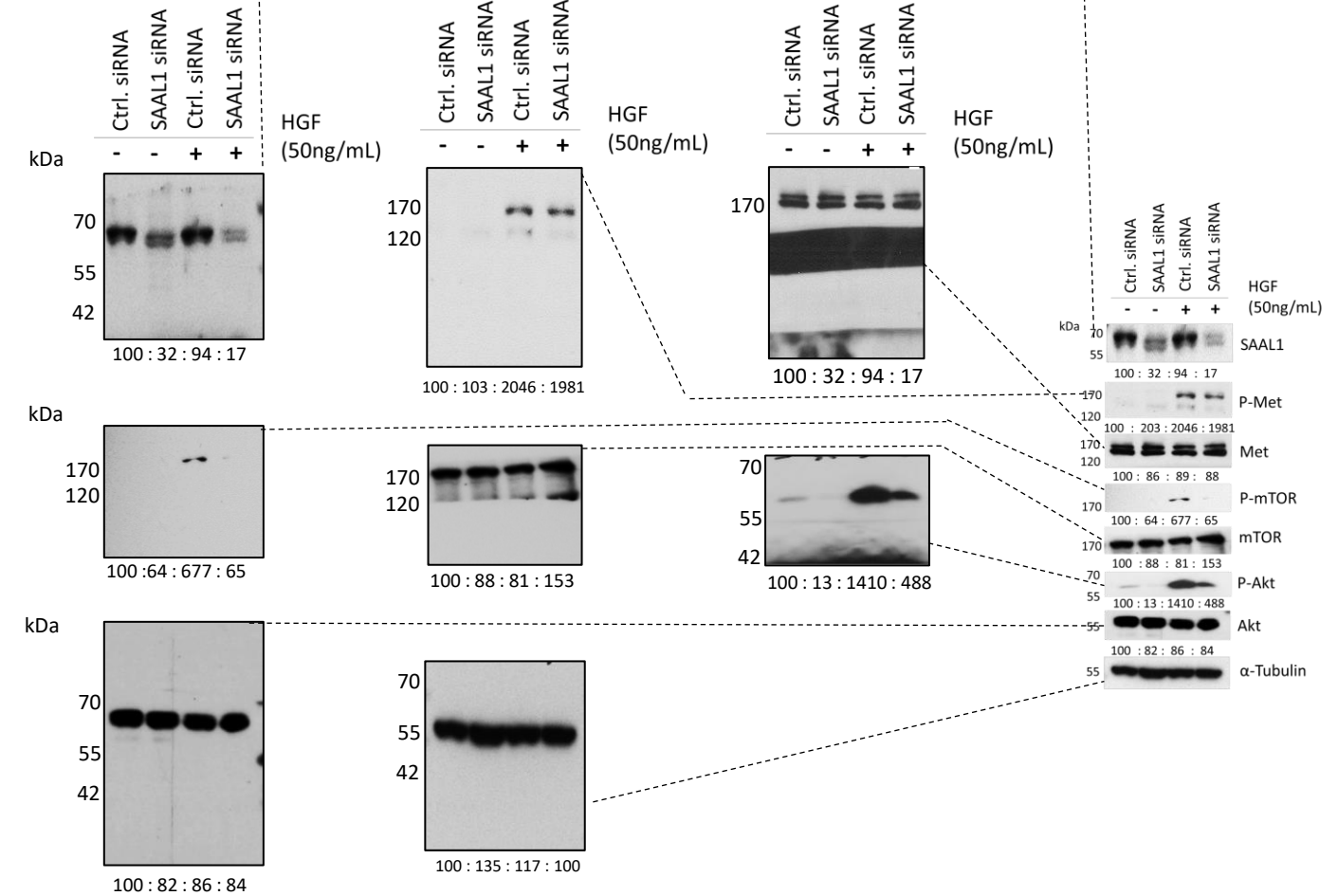

Figure 4B

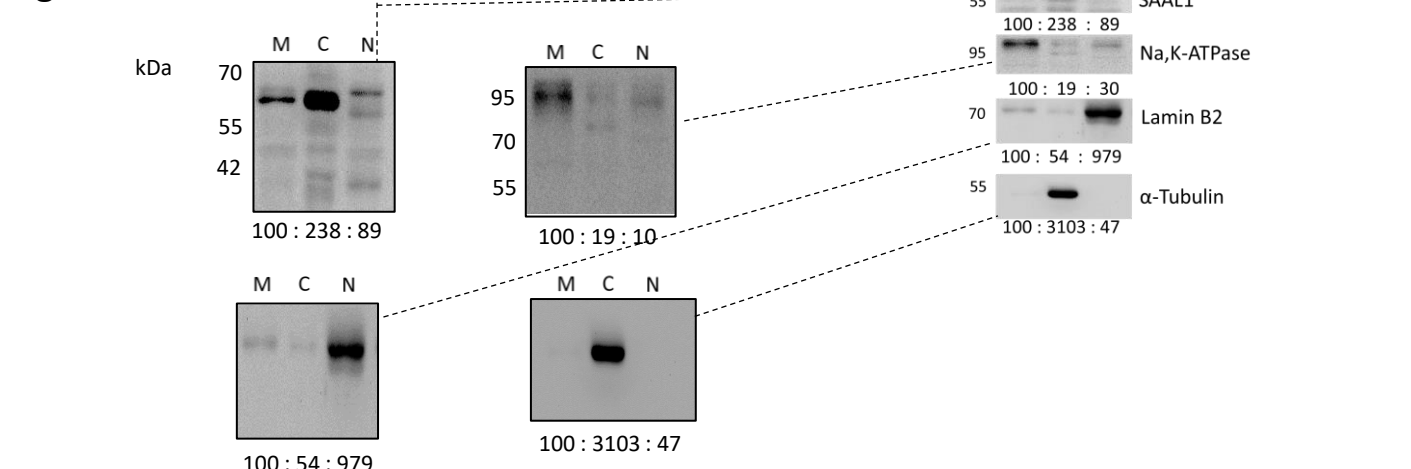

Supplement: Supplementary file 1 [file cancers-12-01843-s001.zip › cancers-864954-suppl/original WB figures.pdf]
